# Revealing Intraosseous Blood Flow in the Human Tibia With Ultrasound

**DOI:** 10.1002/jbm4.10543

**Published:** 2021-10-22

**Authors:** Sébastien Salles, Jami Shepherd, Hendrik J. Vos, Guillaume Renaud

**Affiliations:** ^1^ Laboratoire d'Imagerie Biomédicale Sorbonne Université, Centre National de la Recherche Scientifique (CNRS) Unités Mixtes de Recherche (UMR) 7371, Institut National de la Santé et de la Recherche Médicale (INSERM) UMR S 1146 Paris France; ^2^ Dodd‐Walls Centre for Photonic and Quantum Technologies, Department of Physics University of Auckland Auckland New Zealand; ^3^ Department of Cardiology Erasmus MC University Medical Center Rotterdam The Netherlands; ^4^ Department of Imaging Physics Delft University of Technology Delft The Netherlands

**Keywords:** MUSCULOSKELETAL DISEASES, BONE ULTRASOUND, ANALYSIS/QUANTITATION OF BONE, RADIOLOGY, AGING

## Abstract

Intraosseous blood circulation is thought to have a critical role in bone growth and remodeling, fracture healing, and bone disorders. However, it is rarely considered in clinical practice because of the absence of a suitable noninvasive in vivo measurement technique. In this work, we assessed blood perfusion in tibial cortical bone simultaneously with blood flow in the superficial femoral artery with ultrasound imaging in five healthy volunteers. After suppression of stationary signal with singular‐value‐decomposition, pulsatile blood flow in cortical bone tissue is revealed, following the heart rate measured in the femoral artery. Using a method combining transverse oscillations and phase‐based motion estimation, 2D vector flow was obtained in the cortex of the tibia. After spatial averaging over the cortex, the peak blood velocity along the long axis of the tibia was measured at four times larger than the peak blood velocity across the bone cortex. This suggests that blood flow in central (Haversian) canals is larger than in perforating (Volkmann's) canals, as expected from the intracortical vascular organization in humans. The peak blood velocity indicates a flow from the endosteum to the periosteum and from the heart to the foot for all subjects. Because aging and the development of bone disorders are thought to modify the direction and velocity of intracortical blood flow, their quantification is crucial. This work reports for the first time an in vivo quantification of the direction and velocity of blood flow in human cortical bone. © 2021 The Authors. *JBMR Plus* published by Wiley Periodicals LLC on behalf of American Society for Bone and Mineral Research.

## Introduction

Based on studies with animal models, intraosseous blood circulation is considered to play a key role in bone growth and remodeling, bone metabolism, fracture healing, osteointegration of bone scaffold, the development of bone disorders and metastasis, and joint diseases.^(^ ^1^, ^2^, ^3^, ^4^, ^5^, ^6^, ^7^, ^8^, ^9^, ^10^ ^)^ Yet our knowledge of intraosseous blood flow remains extremely scarce compared to other organs, because of a lack of suitable and accurate noninvasive methods for its in vivo quantification.

In animals, the microsphere technique is generally accepted as the “gold standard” for measuring blood perfusion in bone.^(^ ^11^ ^)^ It is invasive, as it requires sampling of tissue and embolization of capillaries, and is therefore inapplicable in humans.

The in vivo assessment of intraosseous blood flow in humans has been attempted with several techniques. Dynamic contrast‐enhanced magnetic resonance imaging (DCE‐MRI) and diffusion‐weighted MRI have been proposed to assess marrow perfusion in trabecular bone and were limited to rather large regions of interest (vertebra, femoral head).^(^ ^12^, ^13^, ^14^ ^)^ Recently, Wan and colleagues^(^ ^15^ ^)^ showed that DCE‐MRI can assess cortical bone perfusion. Dynamic positron emission tomography (D‐PET) uses ionizing radiation, and its low spatial resolution (about 5 mm) does not allow the clear distinction between blood flow in cortical bone, marrow, and soft tissues surrounding bone. Even if DCE‐MRI and D‐PET can provide an absolute estimation of the time‐averaged blood flow rate, the acquisition time is long (up to 1 hour); the outcome relies on tracer kinetic modeling and the measurement of an arterial input function. Researchers often choose to measure semiquantitative parameters that do not provide an absolute measure of blood flow.^(^ ^13^, ^16^ ^)^ Near‐infrared optical flowmetry^(^ ^5^ ^)^ (laser Doppler and photoplethysmography) has also been applied to the assessment of intraosseous blood flow, but it is limited to an investigation depth of about 1 cm and, like PET, has low spatial specificity.^(^ ^17^ ^)^ Although optical flowmetry achieves excellent temporal resolution and enables the measurement of blood flow pulsatility, it only provides a semiquantitative measurement of blood flow. It is also worth noticing that none of the existing techniques to measure intraosseous blood circulation can determine the direction of blood flow. Moreover, although these techniques have been used for research, they are not used for routine clinical diagnosis of bone perfusion.

Although the relevance of blood flow to bone disorders has been confirmed in animal models, this has not been shown as clearly in humans. DCE‐MRI showed increased contrast agent perfusion in knee osteoarthritis and proximal femur osteoarthritis,^(^ ^18^ ^)^ whereas decreased contrast agent perfusion was observed in proximal femur osteoporosis and spine osteoporosis.^(^ ^13^, ^19^ ^)^ D‐PET with radioactive water (^15^O‐water) evidenced increased time‐averaged blood flow rate in the osteoarthritic femoral head^(^ ^20^ ^)^ and a 14‐fold increase in tibial blood flow 2 weeks after fracture.^(^ ^16^ ^)^ D‐PET with 18F‐Fluoride demonstrated that skeletal blood flow is associated with the rate of bone remodeling activity.^(^ ^21^ ^)^ Accordingly, elevated intraosseous blood flow was measured in patients with Paget's disease.^(^ ^22^ ^)^

**Fig 1:**
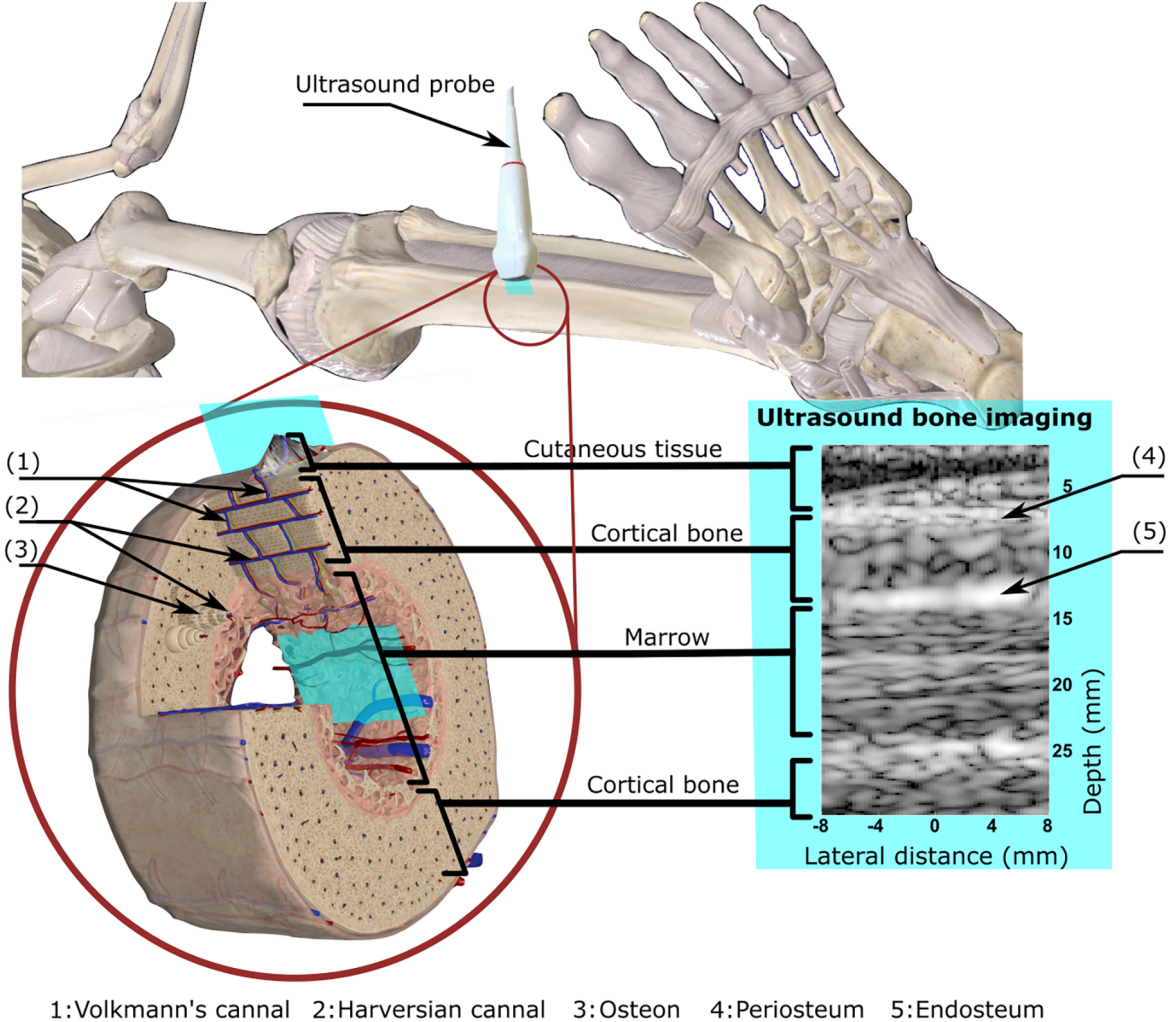
Ultrasound imaging of the bone cortex at the tibia. This figure shows a 3D schematic representation of bones in the leg, the probe positioning on the tibia, the image plane, and the corresponding ultrasound image. The ultrasound probe was placed on the medial surface of the tibia, in the middle of the diaphysis. The blue plane indicates the ultrasound image plane. Three distinct layers are visible in the ultrasound image, namely cutaneous tissue, cortical bone, and the marrow. Haversian canals (2) provide a passage for blood vessels and nerve fibers through the hard bone matrix. In addition, the perforating Volkmann's canals (1) ensure communication between Haversian canals. Blood can therefore circulate between the periosteum and the endosteum. Note that the 3D schematic representation of the vascular organization in the diaphysis of a long bone depicts a cortex with a small thickness (<1 mm, whereas the tibial cortical thickness is about 5 mm). Modified excerpt from Complete Anatomy ’20 with permission from 3D4Medical (www.3d4medical.com). (1): Volkmann's canal; (2): Haversian canal; (3): osteon; (4): periosteum; (5): endosteum.

Ultrasound imaging is relatively inexpensive, does not produce ionizing radiation, offers good spatial resolution and excellent temporal resolution, and has the potential to investigate deeper tissues compared to optical techniques. Ultrasonography provides a direct and absolute measurement of the velocity and direction of blood flow.^(^ ^23^, ^24^ ^)^ Nonetheless, clinical ultrasound scanners currently rely on a major assumption during image reconstruction: the speed of sound in the region of interest is assumed to be uniform. This assumption is valid for soft tissues, but it does not hold for bone. As a result, conventional ultrasonography fails to evaluate blood flow in cortical bone and marrow; only the vascularization of the periosteum (the membrane that covers the outer surface of bones, as shown in Fig. 1) could be assessed.^(^ ^25^, ^26^ ^)^

In this work, we present an approach that overcomes the current limitations of conventional ultrasonography and provides a directional and quantitative measurement of blood flow in human cortical bone using ultrasound imaging. Our approach is evaluated in vivo to achieve, for the first time, a quantitative measurement of blood flow in the diaphyseal cortex of the human tibia.

## Subjects and Methods

This section presents the materials and methods used to measure blood flow in the tibial cortex and in the femoral artery.

### Ultrasound imaging

We used a Vantage 256 ultrasound scanner (Verasonics, Kirkland, WA, USA). With 256 channels in emission and reception, we were able to connect two ultrasound transducers to the ultrasound scanner to image the femoral artery and the tibia simultaneously.

An L7‐4 linear array (ATL Philips, Bothell, WA, USA) was used for imaging the superficial femoral artery and a P4‐1 phased array (ATL Philips) was used for imaging the cortex of the tibia.

### Refraction‐corrected ultrasound imaging of bone

**Fig 2:**
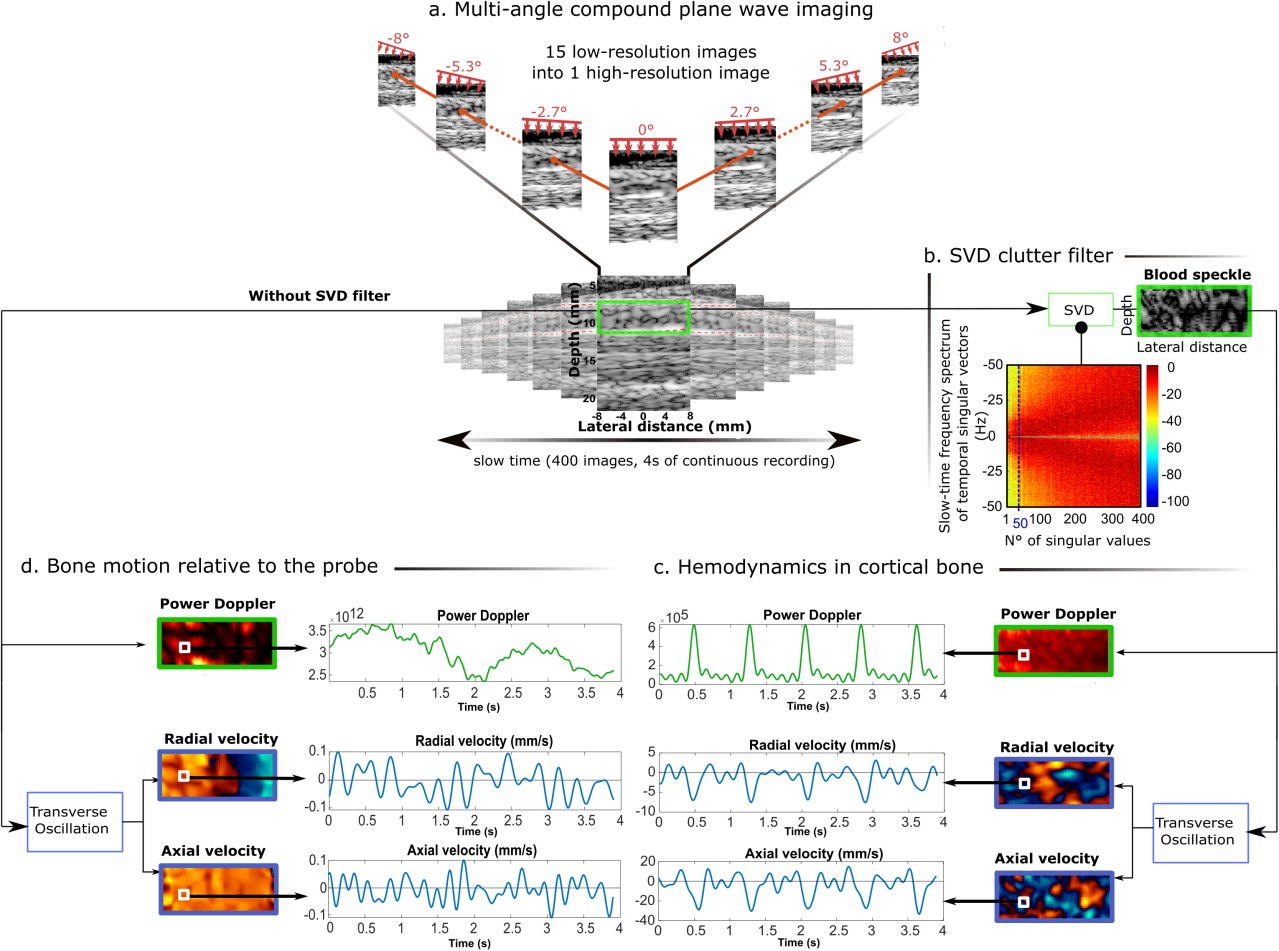
Workflow for the estimation of intraosseous blood perfusion. (*A*) Ultrasound imaging of bone was performed by acquiring 400 compound images at a frame rate of 100 Hz, each frame obtained from 15 planar illuminations tilted from −8 degrees to +8 degrees in the cutaneous tissue. (*B*) The blood signal was extracted from the bone signal by applying a clutter filter based on SVD. (*C*) The power Doppler was calculated from the resulting sequences. Moreover, the axial (long bone axis) and radial (transcortex) components of the blood velocity were estimated using the transverse oscillation approach. (*D*) The same procedure applied to the raw reconstructed images (without SVD filter) shows bone motion relative to the hand‐held probe. The hemodynamic parameters are shown for a single pixel indicated in the images by a white square. SVD = singular value decomposition.

In this work, we used synthetically‐focused ultrasound imaging consisting of a coherent summation of images with low contrast resolution, obtained from different insonification angles, into one image with high contrast resolution. Low‐resolution images can be achieved in different ways, typically by transmitting either plane or spherical waves.^(^ ^27^, ^28^ ^)^ As shown in Fig.  2A, we used the transmission of multiple steered plane waves. Unlike conventional focused‐beam scanning, this technique allows synchronous measurement of blood flow in the entire region of interest.^(^ ^29^ ^)^

In this study, an ultrasound probe was placed on the medial surface of the tibia of volunteers to generate a longitudinal 2D image of the diaphysis. Relying on previous work,^(^ ^30^ ^)^ refraction‐corrected image reconstruction was accomplished. Unlike conventional medical ultrasonography, which assumes a homogeneous medium during image reconstruction, our approach describes the scanned region as a layered medium. Three layers are considered, namely cutaneous tissue, cortical bone, and marrow. The compressional wave‐speed in cortical bone is more than double the wave‐speed in soft tissues. The resulting change in propagation direction (refraction) as ultrasound waves traverse the outer or inner surface of the bone cortex must be taken into account for accurate image reconstruction and blood velocity estimation. In addition, cortical bone exhibits wave‐speed anisotropy that was also accounted for in our reconstruction method using a three‐parameter weak anisotropy model.^(^ ^30^, ^31^ ^)^ The wave‐speed values previously measured in healthy volunteers were used for all healthy volunteers in this new study. More details are provided in the Supplemental Materials and Methods.

Then, after accurate calculation of round‐trip travel times of ultrasound waves, a delay‐and‐sum approach is applied for reconstructing the images.

### Blood flow estimation in cortical bone

In order to capture blood flow, the procedure to generate one image of the cortex was repeated 100 times per second, during a continuous examination of 4 seconds. With the ultrasound technique used in this study, we achieved a spatial resolution close to 1.5 mm in the image of the bone cortex. Consequently, the pores and blood vessels in the cortical bone were not resolved in the ultrasound image. Instead, an ultrasound image of the bone cortex shows speckle, as seen in Fig. 1.

During the 4 seconds of an acquisition, the movement of erythrocytes through blood vessels causes temporal fluctuations in the image. However, relative motion between the hand‐held ultrasound probe and the tibia causes small and slow fluctuations of image intensity as well. These blood‐unrelated fluctuations must be removed to allow quantification of blood velocity. In general, blood‐unrelated fluctuations have a velocity smaller than fluctuations caused by blood flow (Fig. 2*C*,*D*) and high spatial correlation. Singular value decomposition (SVD) was used to extract the time‐variant component in the ultrasound image caused by blood flow^(^ ^32^ ^)^(Fig. 2*B*).

Next, blood‐related fluctuations in the image were analyzed to calculate three flow metrics: a flow metrics proportional to the volume of blood in motion (“power Doppler”)^(^ ^33^ ^)^ and the two components of blood velocity in the 2D image; ie, the axial velocity (blood circulating in the direction of the long bone axis) and the radial velocity (transcortex blood circulation in a direction perpendicular to the long‐bone axis). Power Doppler has the advantage of being robust to noise, but it is relatively angle independent^(^ ^33^ ^)^ and provides no absolute measurement of blood flow velocity. Despite their higher sensitivity to noise, several methods are known in the literature for estimating the 2D vector flow mapping.^(^ ^23^, ^24^, ^34^ ^)^ Here, we chose a method combining a phase‐based motion estimation and the transverse oscillation technique^(^ ^35^, ^36^, ^37^ ^)^ to calculate the axial and radial components of blood velocity at all pixels in the ultrasound image (see the Supplemental Materials and Methods for more details).

The 4‐second acquisition procedure was repeated three times with repositioning of the probes on five healthy volunteers. A continuous recording of 4 seconds ensured that at least three cardiac cycles were observed each time. Each subject was laid down on a hospital bed.

### Blood estimation in the superficial femoral artery

To compare the pulsatility of the blood flow measured in tibial cortical bone with that in the main arterial input in the lower leg, the ultrasound examination was performed simultaneously at the tibia and at the superficial femoral artery, using two hand‐held ultrasound probes operated by one research ultrasound system. Flow quantification in the superficial femoral artery was performed with conventional Doppler ultrasound imaging (see the Supplemental Materials and Methods for details).

**Fig 3:**
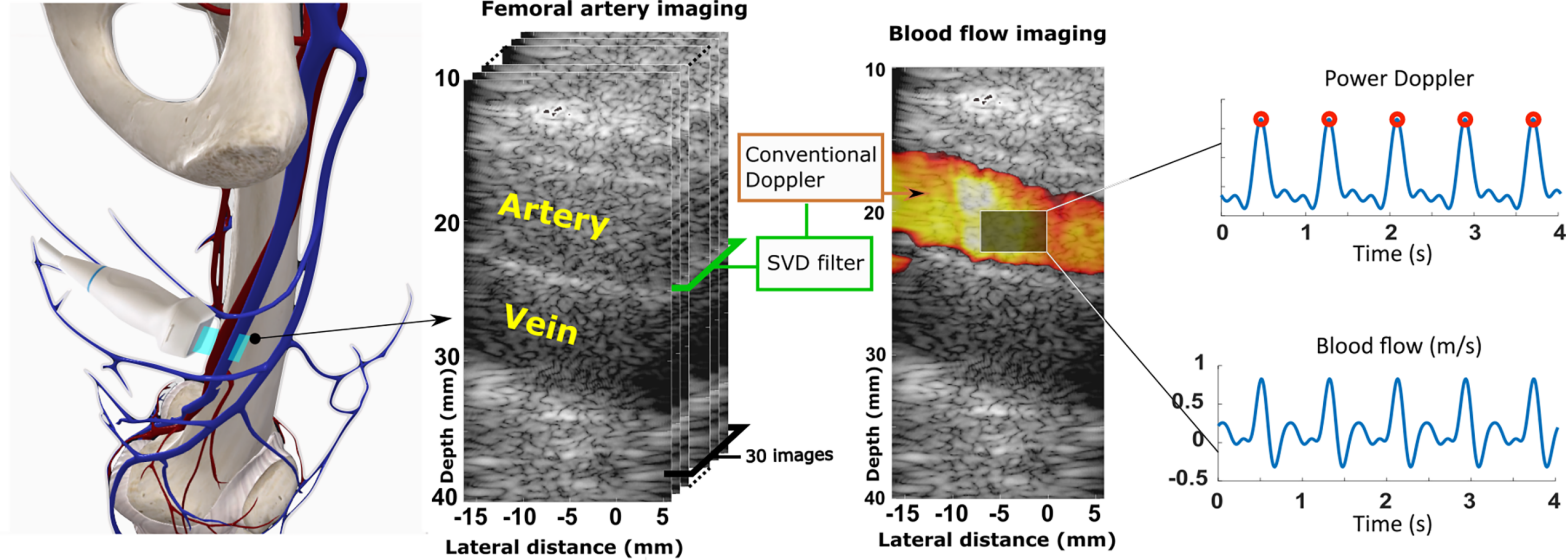
Blood flow imaging in the superficial femoral artery. A total of 400 ultrasound Doppler quantifications were acquired at a rate of 100 Hz. Each quantification was obtained with 30 identical titled (steering angle = 20 degrees) plane‐wave transmissions at a pulse repetition rate of 5000 Hz. After SVD filtering, a conventional Doppler technique was applied to each ensemble of 30 images. The two blue curves show the power Doppler signal and blood flow velocity along the artery's axis. The red circles indicate the power Doppler signal peaks and were used to measure the heart rate. Modified excerpt from Complete Anatomy ’20 with permission from 3D4Medical (www.3d4medical.com).

Peak flow velocity and heart rate were assessed in the femoral artery (Fig. 3) for comparison with the periodicity of blood flow estimated in tibial cortical bone. Because the power Doppler signal is more robust to noise, we used this hemodynamics parameter to estimate the heart rate.

### Human subjects

Five healthy subjects (Table 1) were recruited following approval from the local medical ethics committee of Erasmus MC University Medical Centre Rotterdam, The Netherlands (MEC‐2014‐611). All participants provided written informed consent.

**Table 1:** Subject Age, Total Number of Recorded Cardiac Cycles, and Parameters for SVD Clutter Filtering and Transverse Oscillations

Subject	Age (years)	Total number of cardiac cycles	Method parameters	
			SVD‐filter threshold	Transverse oscillations λ 0× (mm)
1	32	13	50	3.5
2	46	9	40	3.5
3	65	9	50	3.5
4	60	8	40	3.5
5	28	10	30	3.5

## Results

### Hemodynamics measured in tibial cortical bone

The measured bone displacement with respect to the probe was inferior to 0.5 mm, with a peak velocity close to 0.1 mm/s, for all subjects and showed no pulsatility (Fig. 2*D*). After SVD filtering of these slow fluctuations caused by relative probe‐bone motion, the results revealed pulsatile blood flow in cortical bone tissue. Figure 2*C* shows that reproducible pulsatility was observed in all hemodynamic parameters at a given image pixel, namely power Doppler, axial velocity, and radial velocity.

**Fig 4:**
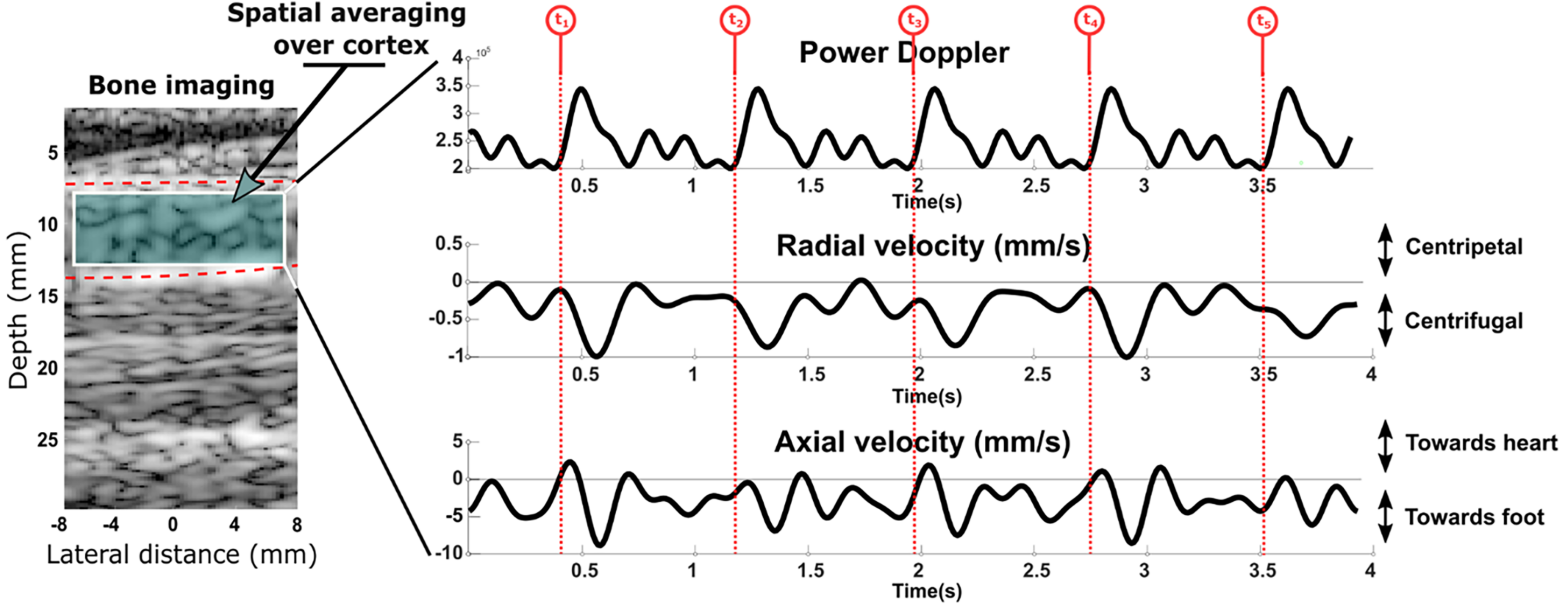
Blood flow in tibial cortical bone measured in subject 1, after spatial averaging over the bone cortex. Pulsatile blood flow in cortical bone tissue is visible in each hemodynamic parameter, namely the power Doppler and the axial and radial velocities. Similar velocity waveforms were observed at the peak flow within five consecutive cardiac cycles (t1, t2, t3, t4, and t5).

**Fig 5:**
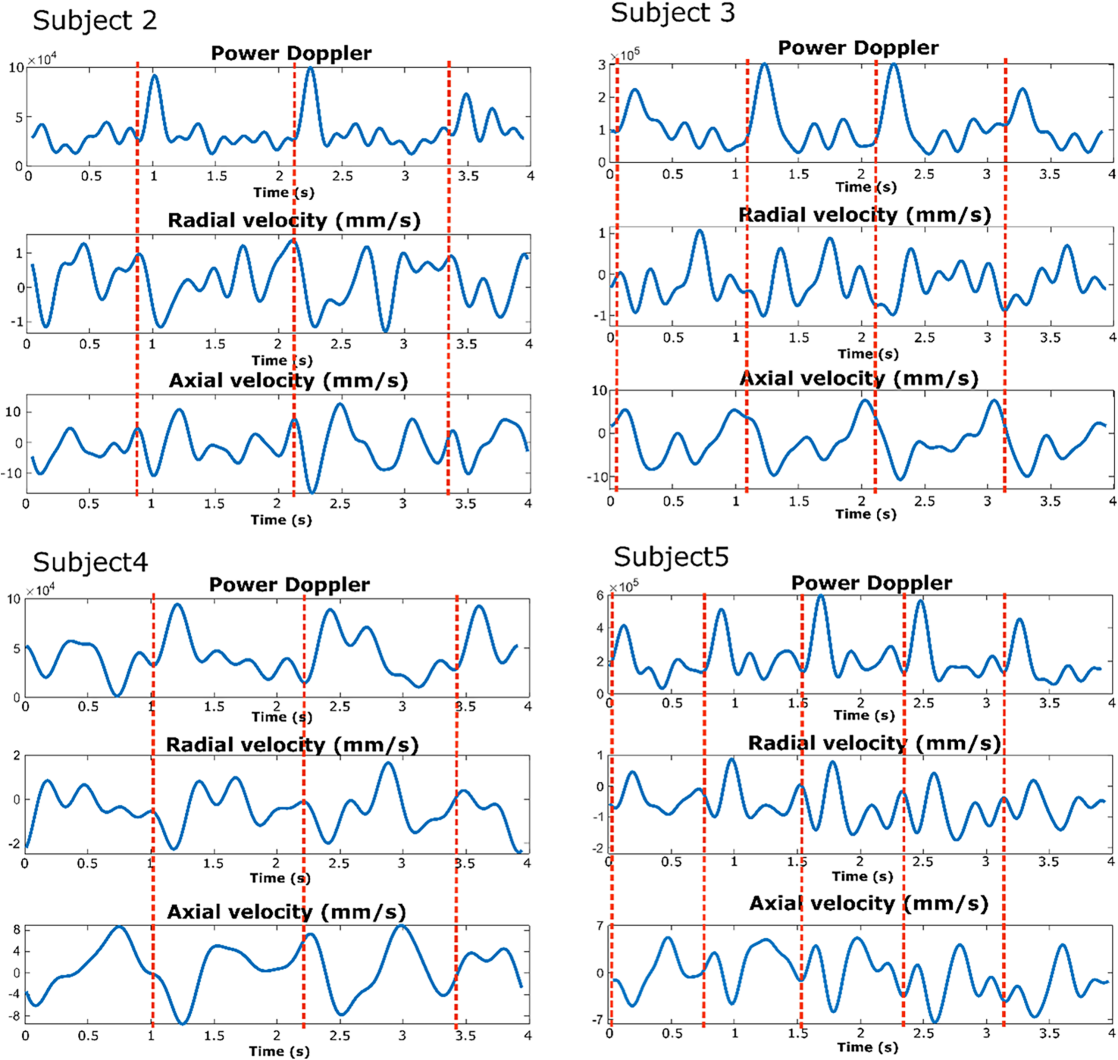
Examples of blood flow measurements in tibial cortical bone obtained in subjects 2–5, after spatial averaging over the bone cortex. Pulsatile blood flow in cortical bone tissue is visible in each hemodynamic parameter, namely the power Doppler and the axial and radial velocities. The red dashed line indicates the cardiac cycles.

**Fig 6:**
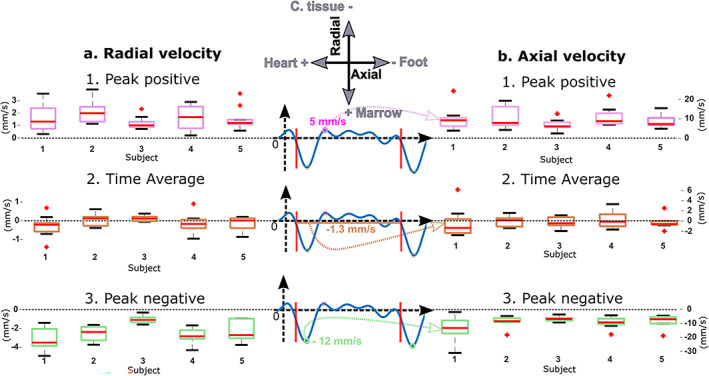
Blood velocity measured in tibial cortical bone for the five subjects. The left column (*A*) shows the radial blood velocity corresponding to blood flow in Volkmann's canals. The right column (*B*) shows the axial velocity corresponding to flow circulating in the Haversian canals. Both columns include the peak positive, peak negative, and time‐averaged values estimated in multiple cardiac cycles for each volunteer. The middle column shows an example of the axial velocity. The axial (radial) peak positive velocity was found between 5.7 (1.3) mm/s and 9.4 (2) mm/s, and the peak negative velocity between −13.9 (−3) mm/s and −7.7 (−1) mm/s, respectively. In each box, the central mark indicates the median, red crosses indicate outlier values, and the bottom and top edges of the box indicate the 25th and 75th percentiles, respectively. The three identical signals in the middle column are used as an illustration to define the quantification parameters. The axial and radial velocity parameter values are also shown in Tables 3 and 4, respectively.

The ultrasound image did not resolve the small blood vessels in cortical bone, and cortical bone possesses a rather organized vascularization with two principal flow directions (Haversian and Volkmann's canals). Because of these two facts, we proposed to spatially average the blood‐velocity vector field over the investigated region of cortex (Fig. 4), which corresponds to a volume of 5 mm × 15 mm × 10 mm (out‐of‐plane image thickness). Interestingly, after spatial averaging over the cortex, pulsatile perfusion was observed in tibial cortical bone in all five subjects (Figs. 4 and 5). Table 2 and Fig. 6 summarize the quantitative flow metrics measured for the five subjects. The peak blood velocity in the direction of the tibia axis (axial velocity) was four to five times larger than that across the cortex (radial velocity). For all five subjects, the largest blood motion corresponded to blood circulating from the medullary cavity to the periosteum (ie, centrifugal flow, from the marrow to the .outside of the tibia, see Figs. 6A1 and 6A3), and from the heart to the foot (Figs. 6B1 and 6B3).

**Table 2:** The Peak Negative, Peak Positive, and Temporally Averaged Values of the Axial, and Radial Blood Velocity in Tibial Cortical Bone Averaged over the Five Subjects

Blood velocity (mm/s)	Average over five subjects	SD
Axial negative peak (long‐bone axis, toward foot)	−9.9	2.3
Axial positive peak (long‐bone axis, toward the heart)	7.9	1.6
Axial time average (long‐bone axis)	−0.4	0.3
Radial negative peak (centrifugal transcortex flow)	−2.3	0.7
Radial positive peak (centripetal transcortex flow)	1.6	0.3
Radial time average (transcortex flow)	−0.1	0.1

The sign convention is depicted in Fig. 4.

The time‐averaged radial and axial blood velocities measured in cortical bone over the 4 seconds of recording (Table 2 and Figs. 6*A*2, 6B2) are small (less than 1 mm/s) and show large variability. Therefore no physiological interpretation of these flow metrics can be made. The smallest negative peak blood velocities were observed in volunteer 3 (Figs. 6*A*3, 6B3, Tables 3 and 4); the axial and radial peak velocities reached −7.7 mm/s and − 1.1 mm/s, respectively (averaged over 12 heartbeats). The largest negative peak blood velocities were observed in volunteer 1 (Figs. 6*A*3, 6B3, Tables 3 and 4); the axial and radial peak velocities reached −13.9 mm/s and −3.0 mm/s, respectively (averaged over nine heartbeats).

**Table 3:** Axial Velocity Measurements of Blood Perfusion in the Tibial Cortex

	Axial velocity (mm/s)					
	Peak positive		Time‐average		Peak negative	
Subject	Average	SD	Average	SD	Average	SD
1	8.0	4.5	−0.8	2.4	−13.9	7.2
2	9.4	5.0	−0.3	1.0	−9.4	3.6
3	5.7	2.5	−0.4	1.0	−7.7	1.5
4	9.3	4.4	0.02	1.6	−9.9	3.4
5	7.1	3.0	−0.5	1.1	−8.9	3.7

**Table 4:** Radial Velocity Measurements of Blood Perfusion in the Tibial Cortex

	Radial velocity (mm/s)					
	Peak positive		Time‐average		Peak negative	
Subject	Average	SD	Average	SD	Average	SD
1	1.6	0.8	−0.3	0.5	−3.0	1.0
2	2.0	0.7	0.02	0.3	−2.4	0.7
3	1.3	0.4	0.1	0.2	−1.1	0.3
4	1.6	0.8	−0.2	0.5	−2.6	0.7
5	1.5	0.7	−0.2	0.4	−2.3	1.0

The flow pulsatility frequency in cortical bone was measured very close to the heart rate observed in the superficial femoral artery, as expected (Table 5). However, no correlation was obtained between the flow peak velocity in the femoral artery and in cortical bone (data not shown).

**Table 5:** Heart Rate Measured With the Power Doppler Signal in the Superficial Femoral Artery and in Tibial Cortical Bone

Subject	Acquisition	Cardiac rate (beat/min)	
		Femoral artery	Cortical bone
1	1	71	73
	2	76	76
	3	71	68
2	1	48	48
	2	47	48
	3	42	43
3	1	64	63
	2	63	63
	3	64	69
4	1	47	47
	2	44	46
	3	48	49
5	1	70	72
	2	73	73
	3	72	72

## Discussion

### Comparison with current knowledge of microporosity of human cortical bone and microvascular flow in other organs

In more than 90% of the cases, the human tibia has one nutrient artery, which penetrates the cortex through a nutrient foramen located below the tibial plateau, at the first proximal third of the tibial length.^(^ ^38^ ^)^ In the medullary cavity, the nutrient artery divides into ascending and descending branches. It is generally accepted that the nutrient artery supplies the marrow and the inner two‐thirds of the cortex. The other one‐third of the cortical blood supply is derived from the periosteal vascular system.^(^ ^39^ ^)^ The Haversian canals provide a passage for blood vessels and nerve fibers through the hard bone matrix, along the direction of the long‐bone axis. Most canals contain a single vessel of capillary structure, although wide canals can contain both an arteriole and a venule.^(^ ^7^ ^)^ In addition, the perforating Volkmann's canals (Fig. 1, (1)) enable blood flow and the passage of nerves between the Haversian canals and across the cortex. Human cortical bone contains more Haversian canals than Volkmann's canals.^(^ ^40^ ^)^ In the cortex of the diaphysis of an adult human long bone, the Haversian canals are nearly aligned with the axis of the long bone.^(^ ^41^ ^)^ Therefore blood in human diaphyseal cortical bone is expected to circulate mainly in the direction of the long‐bone axis. The median diameter of Haversian canals in adult human cortical bone was reported between 40 and 100 μm, and the density of Haversian canals is close to 10 pores/mm^2^.^(^ ^42^, ^43^, ^44^ ^)^ Therefore, the median vessel diameter in human adult cortical bone is expected to be smaller than 40 to 100 μm, and the vessel density is expected to be close to 10 vessels/mm^2^. A recent study observed blood vessels in human femoral cortical bone with a diameter of 50 ± 10 μm.^(^ ^45^ ^)^ Older investigations reported intracortical vessels with a diameter of 15 to 30 μm.^(^ ^46^ ^)^ Blood velocity in such small vessels has been investigated with two‐photon laser scanning microscopy in other organs. In the brain of rodents, pulsatile blood flow in small vessels of similar size was measured with a peak velocity from 1 mm/s to 10 mm/s, depending on the vessel diameter.^(^ ^47^ ^)^

Our findings are therefore in good agreement with current knowledge of the organization of the porosity in human cortical bone and blood velocity in capillaries measured in animals in other organs. First, pulsatile blood flow was observed in human cortical bone at the tibia with a pulsatility rate very close to the heart rate measured in the superficial femoral artery. Second, the axial velocity component was found four to five times larger than the radial velocity component, suggesting a blood flow mainly in Haversian canals as expected from the intracortical vascular organization.^(^ ^40^ ^)^ In contrast, tibial cortical bone in mice possesses transcortical capillaries, mainly.^(^ ^45^ ^)^ Next, the axial and radial blood velocities measured in five healthy subjects are in the range expected for microvascular circulation; ie, from 1 to 10 mm/s (Tables 2, 3, 4).^(^ ^48^ ^)^ Finally, reproducible axial and radial velocity peaks were observed for every subject, indicating peak perfusion going from the heart to the foot and from the marrow to the cutaneous tissue (centrifugal flow). The centrifugal direction corroborates observations in animals and humans reported in the literature.^(^ ^49^ ^)^ Our ultrasound examination was performed in the middle of the diaphysis. Interestingly we observed blood flow from the heart to the foot in all five volunteers, perhaps because the nutrient artery systematically enters the tibia at the first proximal third of the tibial length.

The time‐averaged blood perfusion was found to be less than 1 mm/s for both velocity components; therefore, in agreement with perfusion rate measurements performed in animals with the microsphere technique, which suggested a mean blood velocity in the order of 1 mm/s for a purely unidirectional flow.^(^ ^9^, ^50^ ^)^ Recently, time‐averaged blood velocity in the tibial cortex of mice was reported with intravital laser scanning confocal microscopy in the order of 1 mm/s.^(^ ^45^ ^)^ Thus our findings are in fair agreement with measurements reported with other technologies.

### Relevance of the measurement of the velocity and direction of intraosseous blood flow

Even if the small number of subjects in this study does not allow us to infer general conclusions, it is interesting to note that the smallest peak velocity values were observed in the oldest subject (volunteer 3, age 65 years). Based on the observation of cadaveric human long bones of different age, it was proposed that increasingly severe medullary ischemia with age, brought on by atherosclerosis of the marrow vessels, would cause blood supply of the cortex to evolve from a predominantly medullary blood supply to a predominantly periosteal blood supply.^(^ ^49^ ^)^ This evolution was thought to reduce the amount of circulating blood and its speed in the cortex.^(^ ^49^ ^)^

The proposed approach aimed to characterize blood flow in small vessels both in Haversian canals (along the bone axis) and in Volkmann's canals (perpendicular to the bone axis). Thanks to the measurement of the axial and radial blood velocity components, our analysis provides directional information, which might be of interest for better understanding of bone physiopathology.

### Limitations of the proposed approach

The blood velocity was spatially averaged in a volume of tibial cortical bone of approximately 5 mm × 15 mm × 10 mm. The image thickness (10 mm) was determined by the out‐of‐plane width of the ultrasound beam generated by the phased‐array probe used in this study. Clearly, the use of a matrix‐array probe would improve the quantification of blood flow in the cortex because the information on moving blood could also be specified in the third spatial dimension. As a consequence, the hemodynamics parameters were likely underestimated because the true vascular organization deviates from our idealized description, assuming only two nearly perpendicular networks of parallel vessels. Moreover, the wide pores in cortical bone may host a small arteriole and a small venule. Our analysis likely integrates both arterial and venous flows in a resolution cell of approximately 1.5 mm × 1.5 mm in the ultrasound image, which leads to an underestimated measurement of cortical bone blood perfusion. Nonetheless, a pulsatile blood flow is expected in small arterioles only.^(^ ^47^ ^)^ The results presented demonstrate pulsatile blood flow in the tibial cortex, which suggests predominantly arterial blood circulation. This finding is in agreement with the fact that most canals contain a single vessel of capillary structure.^(^ ^7^, ^46^ ^)^

In this work, an average wave‐speed model for cortical bone was used for all subjects. Knowing that the compressional wave‐speed in cortical bone can vary from one subject to another,^(^ ^51^ ^)^ we would expect a maximum error of 5% on the radial velocity component only. The femoral blood velocity assessment was essential to demonstrate that the pulsatility observed in the blood perfusion of cortical bone was trustful. However, due to the 4‐second recording, the simultaneous ultrasound imaging of the femoral artery and bone, and the hardware memory limitation, a frame rate of only 100 images per second has been used, which is the main limitation of the presented study. Indeed, although 100 images/s is enough to estimate a 10 mm/s blood flow, the limited frame rate and image number are not optimal for the SVD clutter filter. The quality and the spatial/temporal resolution of the measurements may be improved by increasing the number of tilted transmissions and the frame rate. The use of ultrasound contrast agents^(^ ^52^ ^)^ may also significantly improve the estimated sensitivity and specificity of the hemodynamic parameters.

The approach presented herein was able to estimate very slow blood velocity (1 mm/s). Such blood velocity has already been estimated in the rat brain with ultrasound imaging through a cranial window.^(^ ^48^ ^)^ The estimation of very slow blood velocities in a human bone was possible because bones are rigid, thus the only limitation is the motion of the bone relative to the probe, in particular out‐of‐plane motion. However, this limitation may be overcome by using a matrix‐array probe that allows 3D motion correction.

### Perspectives

This work reports the measurement of intraosseous blood flow in the tibial cortex at rest in a supine position. A study of variations in intraosseous blood flow before, during, and after exercise would be interesting. As demonstrated with PET, exercise increases intraosseous blood flow.^(^ ^53^, ^54^ ^)^ Such a study will be considered in future work.

This work presents results obtained at the diaphysis of the tibia; nonetheless, it could be easily applied to investigate the vascularization of other long bones such as the femur or radius. An interesting follow‐up study could be the estimation of intraosseous blood circulation at a different location along a long bone to assess the heterogeneity of blood circulation in the cortex, which was demonstrated in animals with the microsphere technique.^(^ ^9^ ^)^ The presented method might also be used to visualize and measure blood circulation in layers overlying and underlying the bone cortex such as the cutaneous tissue, muscle, and marrow, and study the hemodynamic coupling between layers. The developed approach is expected to unlock the in vivo noninvasive assessment of intraosseous blood circulation in humans. Intraosseous ultrasonography could help to gain new knowledge on vascularization‐related bone physiopathological processes. It may help in the early diagnosis of bone diseases, in the monitoring of the effect of drugs or therapeutic treatments that have an action on intraosseous blood circulation, or in the monitoring of fracture healing.

## Authors’ roles

Study design: GR and SS. Preliminary experiments: JS and GR. Data analysis: SS and GR. Data collection: SS, GR and HJV. Writing‐original draft: SS and GR. Editing and revising manuscript content: SS, GR, JS and HJV.

## Code Availability

The code for image reconstruction of the bone cortex is available as supplementary reference material.^(^ ^30^ ^)^ The code for applying the transverse oscillation method is available online at https://www.creatis.insa-lyon.fr/ius-special-issue-2014/ (see also reference.^(^ ^36^ ^)^

### Peer Review

The peer review history for this article is available at https://publons.com/publon/10.1002/jbm4.10543.

## Supporting information

Additional supporting information may be found online in the Supporting Information section.

**Supplemental Fig. S1**. Fourier series selection filtering method. In the top panel, the blue and red curves are obtained before and after filtering, respectively. The five selected frequencies are indicated with red circles in the bottom panel.

**Appendix S1**. Supplemental Materials and Methods

## Data Availability

The authors declare that all data supporting this study's findings are available within the paper and its Supplementary Information. Raw acquired ultrasound data can be made available upon reasonable request, with permission of Erasmus MC, The Netherlands, and Sorbonne Université, France.
