# Supplementary material for: Revealing Intraosseous Blood Flow in the Human Tibia With Ultrasound
Source: JBMR Plus. 2021 Oct 22;5(11):e10543. doi: 10.1002/jbm4.10543 (PMC8567494; doi:10.1002/jbm4.10543)
Supplement: Supplementary file 1 — Supplemental Fig. S1. Fourier series selection filtering method. In the top panel, the blue and red curves are obtained before and after filtering, respectively. The five selected frequencies are indicated with red circles in the bottom panel. [file JBM4-5-e10543-s001.docx]

**Supplementary material**

|  |
| --- |
| *Supplemental Figure 1:* *Fourier series selection filtering method. In the top panel, the blue and red curves are obtained before and after filtering, respectively. The 5 selected frequencies are indicated with red circles in the bottom panel.* |
