# Supplementary material for: Revealing Intraosseous Blood Flow in the Human Tibia With Ultrasound
Source: JBMR Plus. 2021 Oct 22;5(11):e10543. doi: 10.1002/jbm4.10543 (PMC8567494; doi:10.1002/jbm4.10543)
Supplement: Supplementary file 2 — Appendix S1. Supplemental Materials and Methods [file JBM4-5-e10543-s002.docx]

1. Supplemental Materials and Methods
   1. *Ultrasound* imaging

We used a Vantage 256 ultrasound scanner (Verasonics, Kirkland, WA, USA). With 256 channels in emission and reception, we were able to connect two ultrasound transducers to the ultrasound scanner to image the femoral artery and the tibia simultaneously.

For imaging the femoral artery, we used a L7-4 linear array (ATL Phillips) composed of 128 piezoelectric elements spaced with a pitch of 298 μm. The probe has a central ultrasound frequency of 5 MHz. Four hundred ultrasound Doppler images at a frame rate of 100 Hz were acquired. Each of them was obtained with the transmission of 30 identical titled plane waves (with a steering angle of 20°) at a pulse repetition rate of 5000 Hz.

For imaging the tibia, we used a P4-1 phased array (ATL Phillips) made of 96 piezoelectric elements spaced at a pitch of 295 μm. The probe has a central ultrasound frequency of 2.5 MHz. Four hundred high-resolution compound images at 100 Hz were acquired. Each high-resolution image was obtained from 15 planar insonifications tilted from −8° to +8° in the cutaneous tissue (Fig. 2*A*).

- 1. Refraction-corrected ultrasound image of the bone cortex

Intraosseous image reconstruction was based on an adaptation of the conventional delay-and-sum algorithm. The region of interest was regarded as a layered medium, and refraction occurring at the interface between layers was accounted for. Three layers are considered; cutaneous tissue, cortical bone, and marrow. Transmit and receive travel times were calculated with two-point ray tracing using Fermat's principle with Brent's method.^(30)^ The anisotropy of the compressional wave-speed in cortical bone was modeled with a model of weak transverse isotropy proposed by seismologists ^(55)^

__ (1.1)

$\phi$ is the group angle, or acoustic ray angle ($\phi$ = 0° means normal to the bone axis). $V_{P}^{axial}$ and $V_{P}^{radial}$ are the compressional wave-speeds in the direction of the bone axis and normal to it. $\xi$ is an anisotropy shape parameter. In this work, we used the same wave-speed model for all subjects, with $V_{P}^{radial}$ = 3250 m/s, $V_{P}^{axial}$= 4000 m/s, and $\xi$= 1.5. These values were estimated in two healthy volunteers in a previous study.^(30)^ The compressional wave-speed for the cutaneous tissue layer and the marrow layer was 1540 m/s and 1400 m/s, respectively.

The reconstruction algorithm sums the recorded echo signals (i) along the calculated round-trip travel times over the receive aperture of the probe array, and (ii) over the steered plane wave transmissions (coherent compounding).^(27)^

- 1. Measurement of blood flow in tibial cortical bone

The 2D vector field of blood velocity has been estimated in cortical bone using a method combining a Singular Value Decomposition (SVD) filter ^(32)^ and the transverse oscillation approach.^(36)^ The SVD filter was applied to the 400 high-contrast-resolution images. It removed the echo signals backscattered by the solid porous matrix of cortical bone. After the reception of ultrasonic echoes and image reconstruction, we obtain complex ultrasonic images s(x,z,t). After SVD processing, the spatiotemporal sequence corresponding to ultrasonic data can be rewritten as:

__,(1.2)

where S is the reshaped s matrix into the Casorati matrix with dimension (n_x_ × n_y_, n_t_). Here U_i_ and V_i_ are the spatial and temporal singular vectors of the SVD decomposition. $\lambda_{i}$ are the ordered singular values based on${|\lambda}_{i}|,\lambda_{i+1}<\lambda_{i}$.

Then, the blood signal s_blood_ can be extracted using a threshold value n on the number of singular vectors as follows:

__,(1.3)

The SVD threshold value was chosen manually by looking at the corresponding power Doppler results for each subject (Table 1). Even if different SVD threshold values were used from one subject to another, the same SVD threshold has been used for the three acquisitions on the same subject. A significant improvement of the presented method could be using an automatic SVD threshold detection.^(56)^

We calculated the power Doppler value *I* for each pixel as

$I(x,y,t)={|s}_{blood}{\left( x,y,t \right)|}^{2}$ (1.4)

The transverse oscillation (TO) method ^(35–37)^ was used to calculate the axial and radial blood velocity. It aims to artificially introduce oscillations perpendicular to the ultrasound beam axis, i.e. in the direction of the long bone axis, to estimate the motion using a 2D phase-based approach. The s_blood_ image is convolved with the adapted filter to produce the transverse oscillations. In this study, we used the spatial transverse filter given in ^(4)^ made of the multiplication of a Gaussian window G(x) and a sinusoid

$w(x)=G(x)\times cos(\frac{2\pi x}{\lambda_{0x}})$, (1.5)

where x represents the position of the transducer element and λ_0x_ represents the desired TO wavelength, and we have

$G(x)=\frac{1}{\sigma_{x}\sqrt{2\pi}}\times e^{{-x^{2}}/{2\sigma_{x}^{2}}}$, (1.6)

where σ_x_ is the width of the Gaussian, which is chosen to be of the same order as the ultrasound beam frequency spectrum's width. The convolution is performed in the Fourier domain. The 2-D Fourier transform (FT) of the s_blood_ image is multiplied by a mask, Ω(λ_z_, λ_x_) where each line corresponds to the Fourier transform of ω(x):

__ (1.7

Once TO images have been obtained, it becomes possible to estimate the vector motion using the phase-based technique described in (58). This method aims to create complex image blocks based on a 2-D extension of the analytical signal using the Hann approach.^(57)^ Basically, an image block is generated by keeping only one quadrant of its 2-D Fourier spectrum. Two different analytical images are obtained by keeping two different quadrants of the 2-D Fourier spectra. The 2-D motion vector ($V_{axial}$, $V_{lateral}$) between two successive images is then deduced from the phases φ_11_, φ_12_, φ_21_, and φ_22_ according to

__ (1.8)

with

__, (1.9)

where λ_0z_ is the ultrasound beam wavelength of s_blood_ images corresponding to the wavelength of the signal received by the probe, $\phi_{11}$ and $\phi_{12}$ are the phases of two analytical images from the first image, and $\phi_{21}$ and $\phi_{22}$ are the phases of two analytical images from the next consecutive image. Note that the analytical phase images were smoothed in space and time using a moving average filter of 5 time samples (50 ms) and 5 per 5 spatial samples (1.5 mm × 1.5 mm). For further detail on this motion estimation technique, the reader can refer to.^(58)^ In this study, the same λ_0z_ and λ_0x_ values have been used for every subject and acquisition. λ_0z_ was taken equal to 1.3 mm by assuming a speed of sound of 3250 m/s in the cortical bone ^(30, 51)^ and with a central transducer frequency of 2.5 MHz. λ_0x_ was chosen empirically and set to 3.5 mm. Finally, for each image pixel, the radial and axial velocity time curves were filtered with an in-house Fourier series selection method by retaining only the 5 frequency components with the highest amplitude (an example is shown in Supplemental Fig. S1).

- 1. Measurement of blood flow in the superficial femoral artery

Each ensemble of 30 high frame rate images has been filtered using the same SVD approach, but with a threshold value of 4. Then, the conventional Doppler technique was applied to each ensemble. The blood velocity in the direction of the artery axis was calculated using:

__, (1.10)

where $f_{D}$and $f_{0}$ are the “Doppler frequency” and the ultrasound frequency, respectively, $\vec{v}$is the blood velocity, and __ is the speed of sound. The beam-to-flow angle __ was manually determined in B-mode images.

- 1. Heart rate estimation

Because the power Doppler signal is more robust to noise, we used this hemodynamic parameter to estimate the heart rate. The cardiac rate was measured automatically by finding the local maxima of the temporal power Doppler signal.

The cardiac rate varied from one acquisition to another (Table 5). Consequently, 3 average cardiac rates corresponding to the 3 acquisitions have been measured for each subject. Nonetheless, a good correlation was found between the femoral artery and the cortical bone pulsatility rate for each acquisition and subject.

**References**

55. Thomsen L. Weak elastic anisotropy. *Geophysics*. 1986;51(10):1954‐1966. <https://doi.org/10.1190/1.1442051>

56. Baranger J, Arnal B, Perren F, Baud O, Tanter M, Demene C. Adaptive spatiotemporal SVD clutter filtering for ultrafast Doppler imaging using similarity of spatial singular vectors*. IEEE Trans Med Imaging*. 2018;37(7):1574‐1586. <https://doi.org/10.1109/TMI.2018.2789499>.

57. Basarab A, Gueth P, Liebgott H, Delachartre P. Phase‐based block matching applied to motion estimation with unconventional beamforming strategies. *IEEE Trans Ultrason Ferroelect Freq Control*. 2009;56(5):945‐957. <https://doi.org/10.1109/TUFFC.2009.1127>.

58. Liebgott H, Basarab A, Gueth P, Friboulet D, Delachartre P. Transverse oscillations for tissue motion estimation. *Ultrasonics*. 2010;50(6):548‐555. https://doi.org/10.1016/j.ultras.2009.11.001.
